# Emotions and motivation in mathematics education: Where we are today and where we need to go

**DOI:** 10.1007/s11858-022-01463-2

**Published:** 2023-01-18

**Authors:** S. Schukajlow, K. Rakoczy, R. Pekrun

**Affiliations:** 1 Institute of Mathematics Education and Computer Science Education, University of Münster, Henriette-Son-Str. 19, 48149 Münster, Germany; 2 Department of Early Childhood and Teacher Education, University of Giessen, Karl-Glöckner-Str. 21B, 35394 Giessen, Germany; 3 Department of Psychology, University of Essex, Wivenhoe Park, Colchester, CO4 3SQ UK; 4 Institute for Positive Psychology and Education, Australian Catholic University, Sydney, Australia; 5 Department of Psychology, University of Munich, Munich, Germany

## Abstract

Emotions and motivation are important for learning and achievement in mathematics. In this paper, we present an overview of research on students’ emotions and motivation in mathematics. First, we briefly review how early research has developed into the current state-of-the-art and outline the following key characteristics of emotions and motivation: objects, valence, temporal stability (vs. variability), and situational specificity (vs. generality). Second, we summarize major theories in the field (the control-value theory of achievement emotions, expectancy-value theory of achievement-related motivation, self-determination theory of human motivation, and social-cognitive theory of self-efficacy). Third, we present an overview of instructional characteristics that have been shown to foster emotions and motivation. Fourth, we provide an overview of the contributions to the special issue on “Emotions and Motivation in Mathematics Education and Educational Psychology.” Finally, we suggest directions for future research in the field with respect to advancing theory, improving measurement, and considering diversity and inclusion.

## Introduction

If you go to a mathematics classroom and observe students who are working on mathematical problems, you might hear the following student (S) voices. S1 “Solving this problem was really *fun*”; S2 “I asked myself, do I really *need* to solve this problem?”; or S3 “At the beginning, I *was not sure* whether I could solve this problem.” If teachers (T) shared their observations after class, they might make the following statements. T1 “Most students were *engaged* in the problem solving activity”; T2 “Two students were not *interested* in the mathematics lesson at all”; T3 “Some students complained that mathematics is so *boring.”* In these hypothetical examples, the students and teachers are referring to affective experiences that can be linked to emotional and motivational variables, such as enjoyment (S1), boredom (T3), value (S2), self-efficacy expectations (S3), engagement (T1), and interest (T2). To analyze these and other constructs, several theoretical approaches have been developed, and the following are frequently used: control-value theory (CVT), expectancy-value theory (EVT), self-determination theory, social-cognitive theory of self-efficacy, and interest theory.

In the following sections, we present constructs, theories, and results from research on students’ emotions and motivation. Emotions and motivation can be seen as learning outcomes or goals of mathematics instruction. They are also important prerequisites for cognitive outcomes (e.g., knowledge) and can mediate effects of teaching on these outcomes. Because of the diversity of topics in this area, we could not cover all relevant lines of research here. This overview represents a summary of research on emotions and motivation from the perspectives of mathematics education and educational psychology and is aimed at linking the two research perspectives.

One strength of research in mathematics education is a strong orientation towards the content by taking into account the specific characteristics of mathematics as a domain, as well as mathematical topics or tasks to which emotions and motivation refer. Two examples are a study on students’ motivation regarding modelling, word, and intramathematical problems for two different mathematical topics (Krawitz & Schukajlow, 2018) and a study on self-efficacy regarding geometry versus algebra (Street et al., 2022). One strength of research in educational psychology is that it takes a more general perspective by offering research that is clearly linked to theoretical models with a long research tradition in psychology and education (e.g., see the long tradition in research on achievement motivation in Wigfield & Cambria, 2010). Current developments in society (e.g., changes in education because of the COVID-19 pandemic and increased attention to social-racial inequities) and the formulation of sustainable development goals (UNESCO, 2015) support the importance of students’ emotions and motivation.

The aim of this introduction is to provide an overview of how research on emotions and motivation has developed from the early 1990s to the current state-of-the-art, to contribute to the structuring of the field by reviewing key characteristics of emotions and motivation from different theoretical perspectives, and to summarize leading emotion and motivation theories and teaching approaches for fostering emotions and motivation, particularly in mathematics. We also introduce the contributions to this special issue and discuss directions for future research.

## State-of-the-art in research on emotions and motivation

### Early research on emotions and motivation

For a long time, research in mathematics education has focused on cognitive factors (e.g., knowledge), whereas affective (i.e., in a broader sense, all noncognitive) factors were ignored. Early research was limited to addressing affective constructs on a more general level, for example, by assessing indicators of positive or negative attitudes toward mathematics (Leder, 1987) or students’ beliefs about themselves (Schoenfeld, 1983). Emotions were neglected, with research on mathematics anxiety as one exception (Zan et al., 2006). The turning point in research on emotions and motivation in mathematics education was the development of the taxonomy of affect by McLeod (1992), who differentiated beliefs, attitudes, and emotions along the dimensions of intensity and temporal stability. Beliefs were suggested to be the least intense and the most stable. By contrast, emotions were considered to be the most intense and least stable among the affective constructs. Attitudes were anchored between beliefs and emotions concerning these two key characteristics of affect. Importantly, emotions were targeted as a distinct category but restricted to their situational variant, whereas the dispositional part of emotions was largely ignored. Motivation was included as a subcategory of “beliefs about self” (McLeod, 1992, p. 580 ff). In the late 1990s, Middleton and Spanias (1999) described the state of research on motivation(s), which they characterized very broadly as reasons for human behavior, to the phases of toddlerhood: “… like a toddler, it seems to be going in many directions, frequently getting into trouble” (Middleton & Spanias, 1999, p. 79).

### Trends in research and a modern view on concepts of emotions and motivation

Since the middle of the decade of the 2000s, interest in affect in mathematics education research, including motivation and emotion, has increased. This trend is reflected in Research Forums and Discussion Groups at PME Conferences, the founding of the Topic Study Group “Affect and Teaching and Learning of Mathematics” at CERME conferences, and the publishing of a special issue on affect in *Educational Studies in Mathematics* (Zan et al., 2006). An exemplary analysis of papers published in *Journal for Research in Mathematics (JRME)* and *Educational Studies in Mathematics (ESM)* from 2002 to 2014 indicated an increasing percentage of publications on emotions and motivation in these two mathematics education journals up to 17% in 2014 (Schukajlow et al., 2017), even though the relative number of studies on emotions remained very small (less than 4% in 2014).

To update the analysis, we analyzed all 95 original contributions published in 2021 (77 and 18 articles published in *ESM* and *JRME*, respectively) by using the same coding procedure. A trained master’s student coded the contributions. The first author of this paper then checked the codes for accuracy. The analysis revealed that 41% of the contributions addressed emotions or motivation in their studies, 4% addressed emotions, 18% motivation, and 19% both emotions and motivation. Many of these studies included cognitive variables (e.g., analyses of solution processes or performance tests). The surprisingly high percentage of papers that addressed affect indicated a significant increase in interest in emotions and motivation. One reason for this unexpected increase might be global changes that make the importance of social and emotional learning goals more obvious, such as the COVID-19 pandemic and a stronger focus on issues of social and racial equity. Both topics received more attention in mathematics education recently, which is reflected in an open call in *ESM* for contributions on the COVID-19 pandemic in 2020. This call resulted in an unexpectedly high number of submissions and papers published on the topic of the COVID-19 pandemic in 2021 in *ESM* (Chan et al., 2021).

Concepts of emotions and motivation are defined very differently across authors and fields of research (Hannula et al., 2019; Pekrun, 2021b). The diversity of approaches to emotions and motivation has enriched our understanding of affect. However, many of the diverse emotional and motivational constructs that have been proposed show conceptual overlap. For example, interest is closely related to intrinsic value in EVTs (Eccles & Wigfield, 2020), enjoyment is an important part of the emotional aspects of the person-object theory of interest (Krapp, 2005) and of intrinsic motivation (Ryan & Deci, 2020), and value appraisals are not only antecedents but also components of enjoyment according to the CVT of achievement emotions (cognitive component of enjoyment in Pekrun 2006; Pekrun et al., 2022). This overlap is reflected in the measurement of the constructs. A statement such as “I enjoy doing mathematics” can be used by researchers as an indicator of the enjoyment of mathematics, value of mathematics, interest in mathematics, intrinsic motivation, or as even broader constructs, such as attitude toward mathematics (see more on attitudes in Di Martino & Zan, 2015).

Clarity regarding differences and similarities between constructs on the conceptual and measurement levels is essential for research on emotions and motivation. Recently, Lawson and Robins (2021) discussed how to identify and deal with sibling constructs. The departure point for their considerations was the so-called jingle-jangle fallacy. The “jingle” fallacy occurs when the same construct has different labels. The “jangle” fallacy arises when different constructs have the same label. Because of the large number of different motivational and emotional constructs used in the field, we see a clear threat for jingle-jangle fallacies to occur (Marsh et al., 2019). Typical approaches for dealing with sibling constructs are focusing on one sibling and ignoring the other (i.e., ignoring its relationship with other constructs and theories), acknowledging both siblings in theory and discussion (e.g., by indicating that an increase in interest might be interpreted as an increase in related constructs, such as intrinsic motivation or value), combining the two siblings in an overarching construct, or using statistical methods (i.e., controlling for the joint variance of siblings) to identify the unique contribution of the specific construct.

Even though controlling for related constructs has its strengths, one limitation is that the breadth of the target construct may be lost due to the partialling out of important components (Lawson & Robins, 2021). For example, what would a scale on self-efficacy measure when academic self-concept is controlled for? Performing two types of analysis (i.e., one controlling for the joint variance and one not) might be a way to collect information about the robustness of the analysis. This information can help to avoid artefacts in the interpretation of results.

As motivational and emotional constructs build complex systems within an individual or group of individuals, changes in one motivational variable within a person can affect other motivational variables and emotions in the same individual or in other individuals in complex and nuanced ways. Acknowledging this idea, researchers assume the existence of an “affect system” within an individual or group of individuals (Pepin & Roesken-Winter, 2015, p. xv ff.). Because of complexity in research on emotions and motivation, structuring along key characteristics is essential for improving theories and generating cumulative empirical evidence.

### Structuring the field: characteristics of emotions and motivation

Given the strong overlap and diversity in approaches used to describe and study motivational and emotional processes, it is important to structure the field. Three key characteristics of emotions and motivation are of crucial importance for doing so, namely, valence, temporal stability, and the object (Schukajlow et al., 2017). A new characteristic that has received increased attention recently is the situational nature of affective constructs (Nolen, 2020; Wigfield et al., 2021). Given its importance to the new developments in the field, we decided to include this characteristic in this overview.

*Valence* Valence refers to individuals’ perceptions of desirability or pleasantness. The valence of an affective construct can be positive, negative, or neutral. Positive or negative valence is often used to characterize emotions or attitudes (Di Martino & Zan, 2015; McLeod, 1992; Pekrun, 2006). Importantly, positive (i.e., pleasant) emotional or motivational constructs (e.g., enjoyment or hope) are not always positively related to learning outcomes, and negative (i.e., unpleasant) emotions (e.g., test anxiety) and motivation are not necessarily negatively related to academic achievement in mathematics. In these examples, enjoyment or hope have positive valences because they include pleasant feelings, whereas anxiety has a negative valence because it includes unpleasant feelings. Some motivational constructs (e.g., intrinsic motivation) can also be assigned a positive valence, whereas other motivational constructs or beliefs do not have a positive or negative valence by definition. For example, the belief that mathematics is a science of procedures is not per se related to desirability or pleasantness.

*Temporal stability* Temporal stability versus variability has been acknowledged for a long time to be an important characteristic of affect in psychology (Cattell, 1943; Eysenck, 1987) and later also in mathematics education (McLeod, 1992; Hannula, 2012) underlined the importance of this characteristic and made a call to distinguish between theories that focus on more temporally stable aspects (i.e., traits) and less temporally stable aspects (i.e., states). Indeed, some theories focus mostly on states, such as the flow theory (Csikszentmihalyi, 1997), whereas other theories include state and trait components, such as situational and individual interest in theories of interest (Hidi & Renninger, 2006; Krapp, 2005), or situational versus generalized confidence in performing specific tasks in the case of self-efficacy expectations (Bandura, 2003). Temporal stability is closely related to the situational nature of emotions and motivation. Situational interest in solving a problem, or self-efficacy expectations, often change not only dynamically over time but depend on the situational context. By definition, states can vary across different situations. By contrast, traits can be either generalized across types of situations (e.g., general trait anxiety) or situation- or domain-specific (e.g., trait mathematics anxiety).

*Situational specificity* Situational characteristics of emotions and motivation refer to their embeddedness in tasks, social environments, and sociocultural contexts. Social- cultural practices have repeatedly been emphasized in the context of mathematical learning (Hannula, 2012), such as the role of social-cultural norms for students’ expectations in problem solving (Yackel & Cobb, 1996). Situational approaches to motivational constructs underline the embeddedness of the individual in the social-cultural environment (Nolen, 2020; Nolen et al., 2015; Wigfield et al., 2021). One of the central aims of situational approaches is to account for interactions between individual and social-cultural factors in specific situations. Situational factors in the generation and development of emotions and motivation are an essential part of models of emotions and motivation. Pekrun’s (2006, 2021a) CVT specifies how social environments influence students’ emotions by shaping their control and value appraisals. Eccles’s EVT (Eccles & Wigfield, 2020) includes the cultural milieu as a predictor of individuals’ perceptions, beliefs, achievement expectations, and achievement behavior. Self-determination theory considers competence, autonomy, and social relatedness as three basic needs that can be satisfied as a result of students’ interactions with the learning context (Ryan & Deci, 2020). Taking into account the nature of the situation in research on emotions and motivation might help overcome the marginalization of student groups or individuals (Nolen et al., 2015) by considering social norms (e.g., from right/wrong to more/less appropriate mathematical models/results) and building new student identities in the classroom (e.g., students who can pose a meaningful problem).

**Fig. 1:**
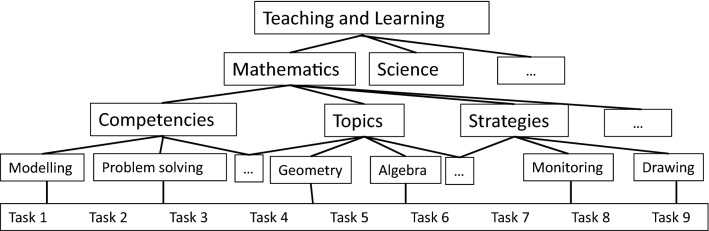
Objects in mathematics education

*Objects* Emotions and motivation are focused on different objects. Objects are addressed as important parts of theories on emotions and motivation. In Pekrun’s (2006, 2021a) CVT, achievement emotions are classified into the following two groups on the basis of their objects: emotions related to achievement activities (activity emotions, e.g., enjoyment and boredom) and emotions related to success or failure in outcomes of these activities (outcome emotions, e.g., hope and anxiety). In theories of interest, researchers emphasize the importance of the object of interest (Hidi & Renninger, 2006; Krapp, 2005). We assume a hierarchical structure of objects in teaching and learning (Fig. 1). On the highest hierarchical level, the object in education is teaching and learning, followed by the teaching and learning of mathematics as a domain. Mathematical teaching and learning are followed by categories, such as competencies, topics, strategies, and others, all of which are specified on the next lower level, for example, as modelling competencies or problem solving competencies, mathematical topics (e.g., geometry or algebra), and strategies (e.g., the monitoring strategy or drawing strategy). Specific mathematical tasks are located on the most basic and concrete level where researchers might address emotions or motivation regarding the solving of specific problems, such as the Monty Hall problem (Krauss & Wang, 2003). We argue that it is essential to be clear about the object of emotions and motivation in both theoretical models and empirical research, as results might be greatly dependent on the type of object. If emotions or motivation that emerge during mathematics lessons focus on nonmathematical content as an object, they can have different effects compared with emotions and motivation that are related to mathematical tasks. For example, female students’ enjoyment of and interest in fashion was found to result in lower engagement in mathematics and prevent them from solving word problems (Boaler, 1994).

Many prior studies have addressed mathematics as an object of emotions and motivation in questionnaires by using a description of mathematical activities in the assessment of emotions (Bieleke et al., 2022), interest (Frenzel et al., 2012), or motivation, or by using specific tasks, for example, to define self-efficacy expectations (Hackett & Betz, 1989; Street et al., 2017) or in experience-sampling studies. Some recent studies have also analyzed other objects, including competencies, mathematical practices, topics, or strategies (Krawitz & Schukajlow, 2018; Nuutila et al., 2020; Siefer et al., 2020; Ufer et al., 2017). In one study, researchers assessed indicators of the hierarchical structure of objects of enjoyment, from enjoyment of life via context- and situation-specific experiences of enjoyment to activity-specific experiences of enjoyment, such as the enjoyment of mathematical tasks (Goetz et al., 2006).

Importantly, objects of emotions and motivation are often complex in nature and include different aspects. For example, Schukajlow et al. (2021a, b) analyzed strategy-based emotions and motivation and their relationships with the quantity and quality of drawing strategies and students’ performance. The emotional and motivational constructs were enjoyment, anxiety, self-efficacy, value, and cost regarding the use of the drawing strategy while solving geometrical modelling problems. These studies addressed a specific strategy (drawing strategy) and specific mathematical competencies (modelling competencies). The results documented the importance of emotions and motivation for strategy quality, strategy use, and cognitive performance outcomes.

We expect that emotions and motivation regarding more general objects (e.g., enjoying learning mathematics) might be more stable across time and contexts within an individual compared with emotions and motivation regarding more specific objects, such as enjoying using the drawing strategy to solve a given mathematical problem or self-determined motivation while learning about different topics in the classroom (Rakoczy et al., 2022). For research on relationships between emotions, motivation, and cognitive outcomes, a degree of alignment between the objects targeted by outcomes is crucially important. Clearly, it can be expected that constructs that target the same object should be more strongly related than constructs that target different objects (Goetz et al., 2013).

Here, we focused on four universal characteristics of emotions and motivation, namely, *valence, temporal stability* (vs. variability), *situational specificity* (vs. generality), *and objects* of emotions and motivation. These characteristics are addressed again in the final section on future directions.

### Achievement emotions

Different theoretical traditions and research paradigms, such as Darwinian, Freudian, or cognitive-psychological paradigms, have shaped descriptions of emotional experiences and definitions of emotions in the past (Hannula, 2015). Contemporary definitions of emotions include multiple components and emphasize the complexity of emotions by referring to affective, cognitive, physiological, motivational, and expressive parts (Shuman & Scherer, 2014). Depending on the object of emotions, researchers group emotions into epistemic, social, moral, topic-specific, or achievement emotions (Pekrun, 2021a). Epistemic emotions focus on the generation of knowledge, and social and moral emotions focus on other people or moral norms. Achievement emotions are very important in the context of education, as they are closely related to achievement-related activities (e.g., problem solving in a mathematics classroom) or outcomes (e.g., school grades). Outcomes are usually differentiated into past outcomes (e.g., pride about being successful on a mathematics test) and future outcomes (e.g., fear of failure on an upcoming mathematics test).

Research on emotions in education in recent decades—and this special issue is no exception—has been anchored to great extent in CVT. According to CVT (Pekrun, 2006, 2021a; Pekrun et al., 2022), two types of appraisals are of primary importance for the arousal of emotions in achievement-related settings. Control appraisals refer to the degree of controllability of actions and outcomes in a given situation (e.g., confidence in solving mathematical problems in class or on a test). Value appraisals refer to the perceived value of the activity or outcome (e.g., the value of learning mathematics or the importance of mathematics grades). Many studies have empirically confirmed the proposed relationships between control and value appraisals and emotions, including appraisals and emotions in mathematics.

The growing body of studies on emotions has stimulated researchers to conduct meta-analyses on the link between emotions and student achievement. Examples are a meta-analysis of research on activity achievement emotions (Camacho-Morles et al., 2021), emotions in technology-based learning environments (Loderer et al., 2020), and single emotions, such as boredom (Tze et al., 2016) or mathematics anxiety (Barroso et al., 2021). One important characteristic of CVT that has contributed to its popularity is the consideration of domain-specificity, which calls for research on emotions in each domain, including mathematics. Indeed, students’ emotions can differ considerably across different domains. Students who are bored in mathematics might not be bored in Latin, English, or music classes. Further, relationships between emotions (e.g., enjoyment) and learning outcomes seem to be slightly stronger in mathematics than in other domains (Camacho-Morles et al., 2021). Interestingly, the number of studies on emotions in mathematics varies by study context and types of emotions. For example, in the context of technology-based environments (Loderer et al., 2020), researchers in many studies have analyzed students’ anxiety in mathematics. Further, the social-cultural context might be important for emotion research. Studies found different effects of emotions in Germany and the US or Canada.

A systematic analysis of relationships between control and value appraisals and mathematical achievements in different countries using Trends in International Mathematics and Science Study data found that, in some countries, enjoyment and boredom mediated these relationships in expected directions. In other countries, they did not mediate the relationships, and in a few countries, the mediation was in the opposite direction (Tze et al., 2021). For example, in Korea, researchers found a negative association between control and value appraisals and boredom but a positive association between boredom and performance.

As students’ anxiety is often addressed in research on mathematics education (Hannula et al., 2019), we discuss research on this emotion in more detail. Anxiety is a prospective outcome emotion, as it focuses on possible failure in the future. Mathematics state test anxiety occurs before or during a test. Mathematics trait test anxiety is a temporary, stable, habitual emotional disposition to respond with anxiety to test situations in mathematics. In math-trait-test-anxious students, even thinking about taking a mathematical test can trigger fear of failure, nervous feelings, worry, and physiological arousal (e.g., increased heart rate and electrodermal activity).

According to CVT (Pekrun, 2006), anxiety arises when students (a) feel they cannot control the situation, implying that failure might be impossible to avoid (low control appraisals), and (b) ascribe a high level of importance to the exam and possible failure (high value appraisals). Anxiety in mathematics classes is triggered when a student values mathematics classes but thinks they cannot avoid performing poorly in class. If a student does not care what happens in mathematics classes or has a guaranteed way to avoid failure (e.g., by doing homework), there is no reason to be anxious. Anxiety is frequent in achievement settings and occurs often in situations with high demands.

Anxiety is a negative (i.e., unpleasant) emotion. However, this does not mean that it is always negatively related to performance outcomes (Goldin, 2014; Pekrun, 2006). Anxiety can activate the motivation to avoid failure by investing effort. Via avoidance motivation (forms of motivations that are aimed at avoiding undesirable events; Elliot 1999), anxiety can have positive indirect effects on performance. Furthermore, anxiety can facilitate the use of some learning strategies (e.g., rigid rehearsal), which can be helpful under some task conditions. On the other hand, anxiety undermines intrinsic motivation. Furthermore, it reduces working memory resources (attention) through worrying about possible failure, which reduces performance on cognitively demanding tasks that require such resources. Therefore, the overall effects of anxiety on task performance are negative in most students and under most conditions. Like other emotions, anxiety might depend on students’ personal goals and classroom norms (Martínez-Sierra & García-González, 2017).

In line with this view, a recent meta-analysis of relationships between anxiety and motivation in mathematics found that in only one of 73 studies researchers reported a positive (nonsignificant) correlation (Li et al., 2021). Most studies yielded moderate-sized negative correlations between anxiety and motivation (mean *r* = − 0.48 for control appraisals, e.g., self-efficacy; − 0.36 for value appraisals, e.g., interest value). The relationships between mathematics anxiety and grades or test scores were also found to be negative (mean *r*s = − 0.32 in Zhang et al., 2019; − 0.34 in Namkung et al., 2019; and − 0.28 in Barroso et al., 2021). The relationships between mathematics anxiety and cognitive achievement were stronger for Asian compared with European students (Zhang et al., 2019), for more difficult tasks (Namkung et al., 2019), for high-stake tests (Namkung et al., 2019), and for secondary school students compared with primary school students (Barroso et al., 2021; Namkung et al., 2019; Zhang et al., 2019).

However, most of the existing studies used cross-sectional study designs. Although corroborating that anxiety and performance in mathematics are linked, cross-sectional results cannot offer information about the directions of these links. Only a few longitudinal studies have addressed the temporal ordering of mathematics anxiety and performance. In a two-wave path-analytic longitudinal study of seventh to ninth graders, Meece et al. (1990) found that students’ math grades predicted their math anxiety, suggesting that math achievement may drive the development of anxiety beyond any effects of anxiety on achievement. In the analysis by Pekrun et al. (2017), students’ math anxiety was a negative predictor of their math grades across five annual assessments from Grades 5 to 9. In turn, math grades negatively predicted subsequent math anxiety, thus demonstrating that math anxiety and performance can be linked by reciprocal effects over time (see the reciprocal effects model of emotions and achievement in Pekrun et al., 2017).

A few studies have identified the profiles of students who can benefit from mathematics anxiety. For example, in Wang et al.’s (2018) study, students with high math anxiety and high motivation invested greater effort and more time in learning mathematics than students with low math anxiety and high motivation. Personal characteristics, such as mental toughness (i.e., the ability to cope with a stressful situation, see Hasty et al., 2021) or prior knowledge (Schukajlow et al., 2021a), were found to moderate the relationship between anxiety and achievement. In the study by Schukajlow et al., (2021a), anxiety negatively affected modelling performance via the use of the drawing strategy in students with low prior knowledge, whereas the effects were in the opposite direction in students with high prior knowledge.

### Achievement motivation

In a broad understanding, motivation comprises reasons for human behavior and represents psychological forces that shape the goal direction, intensity, and persistence of human behavior (Middleton & Spanias, 1999; Wigfield et al., 2021). Achievement motivation shapes achievement behavior, including task choices and the effort invested in achievement tasks. Today, leading theories of motivation are grounded in social-cognitive approaches (Wigfield et al., 2021). Some of them integrate constructs of basic psychological needs (see more on basic psychological needs below). Prevalent motivational theories used in educational research are Eccles’ EVT (Barron & Hulleman, 2015; Eccles & Wigfield, 2020), self-determination theory (Ryan & Deci, 2020), and self-efficacy theory (Bandura, 2003). On the basis of the significance of the theories in the field, we primarily address the EVT and briefly refer to self-determination theory and the theory of self-efficacy.

Eccles’ EVT (Eccles & Wigfield, 2002, 2020) represents a general framework designed to provide a theoretical foundation for research on achievement motivation. Generally, at the core of EVT’s achievement motivation is the assumption that expectations of success and subjective task values affect achievement-related choices and performance. Expectations of success refer to individuals’ beliefs that they are able to attain success in a given domain or on a given task. As such, these expectations are similar to constructs such as self-concept (*I am good at mathematics*) or self-efficacy (*I am confident that I am able to solve this mathematics problem*). In Eccles’ model, task values comprise four components, namely, intrinsic/interest value (e.g., *I like mathematics*), utility/extrinsic value (e.g., *mathematics is important for my future studies*), attainment value (e.g., *being good at mathematics is part of who I am*), and cost (e.g., *doing mathematics requires too much time and effort*). In the last decade, the cost component received more attention, including in research reported in the contributions to this special issue (Beswick et al., 2022; Jenifer et al., 2022). Originally (Eccles et al., 1983), cost was suggested to consist of effort cost (How much effort is needed to perform a task?), opportunity cost (How much time does performing one task take away from doing alternative tasks?), and emotional cost (anxiety about and the social cost of failure). In some studies, cost was found to be a distinct factor. Cost could be statistically separated from expectancies and task value and was found to predict achievement outcomes in mathematics beyond the other two motivational components (Flake et al., 2015; Jiang et al., 2018; Schukajlow et al., 2021b).

On the basis of these empirical results and theoretical considerations of the complexity of costs, some researchers suggested that cost is a distinct and unique component of motivation and relabeled the original model the expectancy-value-cost model (Barron & Hulleman, 2015). However, Eccles & Wigfield (2020) still distinguished cost as an essential component of task value. They explained the empirical results by referring to the valence of costs. Typically, in line with conceptions of cost as negative value in economic theories or Pekrun’s CVT, intrinsic, extrinsic, and attainment value are conceptualized and measured as having a positive valence, whereas cost is seen as having a negative valence. Simultaneously considering positive and negative sets of values (valuing vs. devaluing) might shed light on the meaningfulness of distinguishing between value and cost (Eccles & Wigfield, 2020). This approach was used in studies on reading (Wigfield et al., 2012), but to the best of our knowledge, has yet to be applied in mathematics.

Another important research direction involves analyzing the unique contributions of each value component to achievement and the roles of interactions between different components of Eccles’ EVT. Importantly, expectancies have repeatedly been found to predict mathematical performance and grades, whereas task value is strongly associated with students’ choices, such as a decision to take an advanced mathematics course in school or to study a STEM subject at university (Meyer et al., 2019; Musu-Gillette et al., 2015; Wigfield & Cambria, 2010). The importance of the type of achievement outcome for research on mathematics motivation is further underscored by findings on the stronger associations that expectancies and values have with grades than with test performance (Meyer et al., 2019).

Many studies have suggested that expectancies and task value components are associated with learning outcomes in mathematics. In a study with sixth graders in England, researchers found positive associations between self-concept, value, and achievement in mathematics (Putwain et al., 2019). However, the association between attainment value and achievement was not significant, after self-concept, attainment value, and their interaction were included in the model as simultaneous predictors of achievement. Similarly, relationships of self-concept, interest value, utility value, attainment value, and cost with grades, final exam scores, and test scores in mathematics, remained significant only for self-concept after self-concept and values were included as simultaneous predictors of achievement (Meyer et al., 2019). Interactions between expectancies and values were found to contribute to achievement in mathematics in the studies by Putwain et al. (2019) and Trautwein et al. (2012) but not in the study by Meyer et al. (2019). There is initial evidence of reciprocal associations between motivation and achievement over time as proposed in Eccles’ EVT. As Eccles and Wigfield (2020) noted, “Today’s choices and performances become tomorrow’s past experience” (p. 3). For example, values were reciprocally associated with mathematics grades in upper secondary school in the study by Weidinger et al. (2020). Further, expectancies and values were found to be related to engagement in inquiry-based learning (Fielding-Wells et al., 2017) and to the complexity and quantity of the problems generated by students in research on problem posing (Guo et al., 2020).

As noted above, social-cultural influences are very important for motivation, a trend that was recently reflected by changing the name of the expectancy-value model to the *situated* expectancy-value model (Eccles & Wigfield, 2020). These influences refer to developmental aspects of motivation. From early on, children interact with parents, other children, and adults in specific situations. Complex interactions between parents’ characteristics and beliefs and the child’s characteristics predict how motivation develops in childhood. More research on these interactions is needed (Eccles & Wigfield, 2020).

*Self-determination theory* is another important theoretical framework with specific relevance for learning and achievement. Self-determination theorists posit the existence of three basic psychological needs (Ryan & Deci, 2020), namely, the needs for autonomy, competence, and relatedness. These needs direct human actions across different areas of life. In education, the need for autonomy can be satisfied, for example, when students can make their own decisions and choose their own actions in accordance with their personal goals, without external pressure. Students experience competence, for example, when they feel mastery and have opportunities to improve their knowledge. The need for relatedness is satisfied when students feel they belong and are connected to others.

The fulfillment of basic needs experienced by students was frequently shown to be positively related to learning outcomes. For example, research has shown that students’ autonomy and competence while solving modelling problems in class was positively related to interest at posttest, and the experience of competence was positively related to performance and procedural knowledge (Achmetli & Schukajlow, 2019; Schukajlow & Krug, 2014). Supporting basic need satisfaction can increase intrinsic motivation, interest, and the internalization of external demands. In this theory, motivation is viewed as a continuum ranging from amotivation via several forms of extrinsic motivation to intrinsic motivation (Ryan & Deci, 2020). The overall positive relationships between intrinsic motivation and learning outcomes were supported by a recent meta-analysis on student motivation (Howard et al., 2021). The relationships between extrinsic forms of motivation (“external,” “introjected,” and “identified” motivation) and outcomes were mixed in this analysis and included weak positive relationships between “external” as well as “introjected” motivation and anxiety.

In *Bandura’s social-cognitive theory*, people are seen as active agents in the social environment who can regulate and adapt their behavior. Central to this theory are self-efficacy expectations, which comprise “the beliefs in one’s capabilities to organize and execute the courses of action required to produce given attainments” (Bandura, 2003, p. 3). According to self-efficacy theory, students with high self-efficacy in mathematics will engage in problem solving more intensely and more often, demonstrate higher persistence and effort, and consequently achieve better learning outcomes, such as higher mathematical performance, grades, or final degrees. Four main sources of self-efficacy were proposed (Bandura, 2003), namely, students’ own (mastery) experiences in engaging with math tasks, vicarious experiences while observing others during a mathematical activity, verbal persuasion from others, and the level of arousal during a mathematical activity. Many studies have found a positive association between self-efficacy and achievement outcomes in mathematics and identified sources of self-efficacy in mathematics (e.g., Schunk & DiBenedetto 2020; Usher et al., 2019).

## Instructional methods for improving students’ emotions and motivation

Identifying instructional methods for improving students’ emotions and motivation is important for research in mathematics education, from both theoretical and practical perspectives. A growing number of systematic reviews, meta-analyses, and overviews have focused on the analysis of multiple aspects of instruction in research on emotions and motivation (Camacho-Morles et al., 2021; Higgins et al., 2019; Lazowski & Hulleman, 2016; Rosenzweig & Wigfield, 2016; Rosenzweig et al., 2022; Savelsbergh et al., 2016). However, the number of overviews focusing on emotions and motivation in mathematics instruction is limited (for an exception, see Hannula et al., 2019). Because intervention studies offer a great deal of information for practice, we focused on this kind of study. We analyzed the contributions addressed in systematic reviews and meta-analyses and selected studies from the domain of mathematics. We included interventions that were aimed at affecting emotions or motivation in mathematics and structured the overview of instructional methods according to the affective variables that were addressed (see Table 1). In some studies, researchers used emotion- and motivation-related variables as item-level indicators of attitudes or affect. In these studies, we interpreted changes in attitudes or affect as changes in the underlying constructs. We grouped the interventions according to the following four groups of outcome variables: (a) emotions; (b) expectancies (including self-efficacy), values, and cost; (c) basic needs; and (d) interest. As some interventions analyzed effects on different affective variables, the same intervention can be found several times in the overview.

**Table 1:** Overview of instructional methods

Emotions and motivation as outcome variables	Interventions in mathematics
Emotions	Instruction in basic need satisfaction, emotional support, and self-regulation (Held & Hascher, 2022) Prompting students to develop multiple solutions (Schukajlow & Rakoczy, 2016) Problem posing (Voica et al., 2020) Teacher-oriented instruction and student-centered teaching method (Durandt et al., 2022; Parhizgar & Liljedahl, 2019; Schukajlow et al., 2012) Offering choices and personalization of computer games (Cordova & Lepper, 1996)
Expectancy, value, and cost	Utility-value intervention from writing an essay about the relevance of mathematics for life (Brisson et al., 2017; Gaspard et al., 2015; Hulleman et al., 2010; Kosovich et al., 2019) Utility-value intervention from evaluating quotes from interviews about the relevance of mathematics (Brisson et al., 2017; Gaspard et al., 2015, 2021) Offering choices and the personalization of word problems (Høgheim & Reber, 2015) Instruction on technology, such as an augmented reality intervention (Chen, 2019; Higgins et al., 2019) Teacher-oriented instruction and student-centered teaching method (Durandt et al., 2022; Parhizgar & Liljedahl, 2019; Schukajlow et al., 2012) Instruction in basic need satisfaction, emotional support, and self-regulation (Brandenberger et al., 2018) Training in strategy use and self-regulation (Marcou & Lerman, 2007; Ramdass & Zimmerman, 2008) Problem posing (Voica et al., 2020) Prompting students to develop multiple solutions (Schukajlow et al., 2019) Instruction in basic need satisfaction, emotional support, and self-regulation (Brandenberger et al., 2018) Formative assessment and feedback (Rakoczy et al., 2019; Ramdass & Zimmerman, 2008)
Basic needs (competence, autonomy, social relatedness) and intrinsic motivation	Offering choices and personalization of computer games (Cordova & Lepper, 1996) Prompting students to develop multiple solutions (Achmetli & Schukajlow, 2019; Schukajlow & Krug, 2014) Process-oriented feedback (Rakoczy et al., 2013)
Interest	Offering task choices and the personalization of word problems (Bernacki & Walkington, 2018; Høgheim & Reber, 2015) Reading comprehension prompts (Krawitz et al., 2022) Utility-value intervention from writing an essay about the relevance of mathematics for life (Hulleman et al., 2010; Kosovich et al., 2019; Liebendörfer & Schukajlow, 2020) Teacher-oriented instruction and student-centered teaching method (Durandt et al., 2022; Parhizgar & Liljedahl, 2019; Schukajlow et al., 2012) Prompting students to develop multiple solutions (Achmetli & Schukajlow, 2019; Schukajlow & Krug, 2014) Process-oriented feedback (Rakoczy et al., 2013)

*Note.* The table depicts the main aspects of the interventions

*Emotions* Intervention studies targeting students’ emotions addressed effects of attribution training (Hamm et al., 2014), changes in value appraisals (Gläser-Zikuda et al., 2005), a control-value intervention (Hoessle et al., 2021), and other kinds of instruction. To the best of our knowledge, no attribution training programs were examined in mathematics, and only a few interventions analyzed the effects of emotions in mathematics classes via changes in value appraisals. In a series of lessons and workshops for teachers and students in mathematics over 2 school years, researchers tested an intervention on basic need satisfaction, emotional support, and self-regulation in low-achieving secondary school students (Held & Hascher, 2022). They assumed that this intervention would affect emotions via control and value appraisals. The analysis revealed no changers in control and value appraisals and consequently no effects on emotions. Positive effects of student-oriented teaching compared with teacher-centered instruction on how much secondary school students enjoyed solving mathematical problems were found after a 10-lesson teaching unit on modelling problems (Schukajlow et al., 2012). The authors explained the effects as positive effects of cooperative teaching methods implemented as part of a student-oriented teaching method. Similar effects were also found in Parhizgar and Liljedahl’s (2019) study, which addressed emotions as part of an attitude questionnaire. Durandt et al. (2022) did not find positive effects of a student-oriented teaching method in engineering students (cooperative group work, training in strategy use, and scaffolding self-regulation with a solution plan) on emotions (e.g., anxiety) about solving modelling problems addressed as part of affect questionnaires. Prompting students to develop multiple solutions was found to increase enjoyment and decrease boredom in class (Schukajlow & Rakoczy, 2016). Problem posing was found to enhance positive emotions and to reduce negative emotions compared with problem solving, indicating that asking prospective teachers to pose problems and present their arguments about possible solutions is a promising type of instruction (Voica et al., 2020).

*Expectancy and value* In the EVT framework, most intervention studies have addressed the relevance of the learning content and researchers have analyzed effects on personal utility value, interest, and achievement-related behavior (Rosenzweig et al., 2022). In this type of intervention, students were usually asked to reflect on the relevance of the learning content on the basis of a researcher-supplied text (Gaspard et al., 2021) or to write an essay explaining the relevance of the mathematical content to their friends or parents (Hulleman et al., 2010). Writing essays on the relevance of mathematics for future life increased perceived utility value in algebra courses, with larger effects in male than in female community college students (Kosovich et al., 2019). Positive effects on utility value were found from personalizing the context of word problems (Høgheim & Reber, 2015). New technologies (e.g., implementation of augmented reality in mathematics instruction) increased motivation and decreased anxiety in lower secondary school students (Chen, 2019). Moreover, a meta-analysis demonstrated overall positive effects of technology (videos, software, and feedback based on the software) on motivation in mathematics (Higgins et al., 2019). Training in cognitive, metacognitive, and—in some studies—also cooperative strategies were found to be promising interventions that affect motivational and achievement-related outcomes. Students’ strategy training was found to improve students’ self-efficacy in arithmetic (Ramdass & Zimmerman, 2008).

No positive effects of student-oriented teaching approaches were found for students’ self-efficacy or values compared with teacher-centered instruction (Durandt et al., 2022; Schukajlow et al., 2012). Prompting students to develop multiple solutions (i.e., applying one vs. two mathematical procedures while solving real-world problems) improved self-efficacy expectations in students with low initial self-efficacy (Schukajlow et al., 2019). In a study of secondary school students, researchers found positive effects of supporting students’ basic need satisfaction, emotional support, and self-regulation on intrinsic motivation but no effects on self-concept in mathematics (Brandenberger et al., 2018). Likewise, a similar intervention did not affect self-concept, self-efficacy, or value (Held & Hascher, 2022; Narciss, 2008) emphasized the self-efficacy-enhancing function of another aspect of instruction, namely, feedback. The impact of feedback and formative assessment in mathematics on self-efficacy was investigated in two intervention studies. Rakoczy et al. (2019) found that process-oriented feedback given by teachers in the context of a formative assessment intervention fostered students’ self-efficacy compared with feedback in the form of grades. Ramdass and Zimmerman (2008) showed a positive effect of monitoring and self-assessment, as another form of formative assessment, on the accuracy of self-efficacy ratings.

*Basic needs* We grouped studies that assessed students’ perceived autonomy, competence, and relatedness into this category. Many studies used self-determination theory as a theoretical framework and analyzed the effects of manipulating basic need satisfaction on emotions and motivation (Brandenberger et al., 2018; Held & Hascher, 2022; Voica et al., 2020). However, only a few studies have analyzed whether students experienced higher basic need satisfaction during the intervention. Primary school students who were offered choices and the personalization of a computer game in arithmetic reported higher competence in playing a mathematical computer game (Cordova & Lepper, 1996). Prompting the development of multiple solutions (i.e., solutions based on different assumptions about missing information in modelling problems) affected students’ perceived satisfaction of autonomy and competence needs (Achmetli & Schukajlow, 2019; Schukajlow & Krug, 2014). Rakoczy et al., (2013) analyzed the impact of process-oriented feedback compared with grades on perceived competence and found that students felt more supported in their need for competence after process-oriented feedback.

*Interest* Offering choices and the personalization of word problems were found to increase interest (Høgheim & Reber, 2015; Howard et al., 2021; Patall et al., 2008) offered students word problems embedded in different interest areas (e.g., movies, music, or gaming) and demonstrated that when students can choose problems from their area of interest, then their situational and individual interest increases. Further, researchers analyzed the effects of the personalization of mathematical content by using word problems for the development of tabular, graphical, and symbolic representations of functions. On the basis of students’ out-of-school interests, standard versions of the context presented in word problems were adjusted to personal interests, such as computer games (Bernacki & Walkington, 2018): “A racing game has a train that weaves through tracks and tunnels and travels at a rate of 2.9 feet per second. The train is currently 100 feet from the start of the course and moving toward the finish line.” Researchers found positive effects of personalization on situational and individual mathematics interest in high school students (Bernacki & Walkington, 2018; Høgheim & Reber, 2015). Further, increasing students’ involvement and ease of comprehension by offering task-specific reading comprehension prompts increased students’ situational interest in solving modelling problems in Germany and Taiwan (Krawitz et al., 2022).

Prompting students to develop multiple solutions for modelling problems affected interest (Achmetli & Schukajlow, 2019; Schukajlow et al., 2019; Schukajlow & Krug, 2014). In two studies involving secondary school students, researchers found positive effects of student-centered teaching on situational interest compared with teacher-oriented instruction (Parhizgar & Liljedahl, 2019; Schukajlow et al., 2012), whereas no effects of this teaching method were found in a study involving engineering students (Durandt et al., 2022). Writing an essay about the relevance of mathematics for life affected not only expectancies and values but also interest in mathematics (Hulleman et al., 2010; Kosovich et al., 2019). A mixed methods study involving preservice teachers documented the importance of deep reflections about applications of mathematics (modelling problems) for the effects of a relevance intervention on interest in mathematics (Liebendörfer & Schukajlow, 2020). Consequently, the quality of the reflections was found to be an important mediator of the effects of the relevance intervention. For process-oriented feedback, there was no total effect on interest, but there was an indirect effect on the development of interest via perceived competence support (Rakoczy et al., 2013). That is, the more competence-supportive the feedback was perceived to be, the more interest students developed.

In this overview, we identified the following types of interventions that aimed to promote students’ emotions and motivation in mathematics: (a) student-centered teaching, (b) offering choices during learning, (c) adjusting the learning content to students’ personal interests, (d) prompting students to develop multiple solutions, (e) utility value interventions, (f) problem posing, (g) instruction in basic need satisfaction, emotional support, and self-regulation, (h) strategy use and self-regulation training, and (i) reading comprehension prompts. For most of these interventions, positive effects on affective variables were found. However, some studies yielded no effects on one or several affective variables, and thus, the findings were not consistent. This lack of consistency may be due to differences between studies in theories, intervention methods, fidelity of the implementation of the intervention, study design, samples, sociocultural contexts, and the measures used to assess outcomes. More intervention research is needed to consolidate the existing evidence, clarify what works, and generate cumulative evidence that is suited to guide practice in mathematics education in evidence-based ways.

## Contributions to this special issue

In the studies documented in this special issue (see Table 2 for an overview), researchers assessed various achievement emotions (Bieleke et al., 2022) and epistemic emotions (Schubert et al., 2022). Further, in some papers, emotions were grouped according to their objects, such as emotions about the self, mathematics, or teachers/classmates (Middleton et al., 2022), or according to valence (e.g., positive and negative emotions in Panero et al., 2022). Math anxiety received specific attention (Jenifer et al., 2022; Putwain & Wood, 2022).

The prevalent motivation theory used in the contributions was EVT (Eccles & Wigfield, 2020), which served as the theoretical framework for the contributions by Beswick et al. (2022), Böswald and Schukajlow (2022), Gaspard et al. (2022), Jenifer et al. (2022), Middleton and Wiezel (2022), and Rach (2022). Other important motivational theories considered in the contributions were self-determination theory and Bandura’s (2003) social-cognitive theory of self-efficacy, which were used simultaneously or separately in the contributions by Hettinger et al. (2022), Rach (2022), Skilling and Stylianides (2022), and Zhang et al. (2022). In other papers (Renninger et al., 2022; Seah, 2022; Skilling & Stylianides, 2022), researchers addressed Hidi and Renninger’s (2006) theory of interest development, a cognitive engagement framework (Skilling & Stylianides, 2022), or a theory of well-being as value fulfilment (Tiberius, 2018) in the contribution by Seah (2022).

The prevalent assessment instruments used in the contributions were questionnaires that have been validated in research on emotions and motivation. Researchers addressed a wide range of emotional and motivational constructs in their contributions. Some of the contributions linked the constructs to achievement outcomes (e.g., test performance or final grades). In Table 2, we grouped the contributions according to the theories they used.

**Table 2:** Overview of the contributions in the special issue

Contribution	Theories	Constructs	Assessment	Participants
*Contributions anchored in Pekrun’s CVT*				
Bieleke, Goetz, Yanagida, Botes, Frenzel, Pekrun	CVT (Pekrun, 2006)	Enjoyment, pride, anger, anxiety, shame, hopelessness, and boredom, motivation, strategies, performance	Self-reports, grades, test scores	Secondary school students, Germany
Putwain, Wood	CVT (Pekrun, 2006)	Anxiety, control and value appraisals, performance	Self-reports, test scores	Grade 5 students, UK
Schubert, Pekrun, Ufer	CVT (Pekrun, 2006)	Control and value appraisals, epistemic emotions, attention, motivation, performance	Self-reports, experimental task performance	Undergraduate university students, Germany
*Contributions anchored in Eccles’ EVT*				
Beswick, Watt, Granziera, Geiger, Fraser	EVT (Eccles & Wigfield, 2020)	Expectancies, values, costs, achievement goals, student-perceived classroom and teacher factors, school well-being, and mathematical engagement	Self-reports	Male primary and low-secondary school students, Australia
Böswald, Schukajlow	EVT (Eccles & Wigfield, 2020) Theory of self-efficacy (Bandura, 2003)	Value, self-efficacy	Self-reports	Preservice teachers, Germany
Gaspard, Parrisius, Nagengast, Trautwein	EVT (Eccles & Wigfield, 2020)	Value, relevance support	Self-reports, grades, test scores	Ninth-grade students, Germany
Jenifer, Levine, Beilock	EVT (Barron & Hulleman, 2015)	Anxiety, expectations, cost	Self-reports, grades	Undergraduate university students, USA
*Contributions anchored in self-determination theory, interest theory, or well-being theory*				
Renninger, Gantt, Lipman	Model of interest development (Hidi & Renninger, 2006)	Interest, achievement	Self-reports, test scores	Undergraduate university students, USA
Seah	Theory of well-being as value fulfilment (Tiberius, 2018)	Values	Self-reports	Grade 3 students, China
Zhang, Yang, Kaiser	Self-determination theory (Ryan & Deci, 2020)	Cognitive engagement, intrinsic and extrinsic motivation, achievement	Self-reports, grades	Secondary school students, China
*Contributions anchored in multiple theories*				
Hettinger, Lazarides, Schiefele	Theory of self-efficacy (Bandura, 2003) Self-determination theory (Ryan & Deci, 2020)	Self-efficacy, emotional support, interest	Self-reports, test scores	Teachers and ninth-grade students, Germany
Middleton, Wiezel, Jansen, Smith	CVT (Pekrun, 2006) EVT (Eccles & Wigfield, 2020)	Self-efficacy, self-regulation, interest, emotions, academic and social support	Self-reports, videos	Secondary school students, US
Panero, Castelli, Di Martino, Sbaragli	CVT (Pekrun, 2006) Theory of self-efficacy (Bandura, 2003)	Attitude components (emotions, self-efficacy)	Self-reports	Preservice teachers, Switzerland
Rach	EVT (Eccles & Wigfield, 2020) Self-determination theory (Ryan & Deci, 2020)	Values, competency, autonomy, effort	Self-reports, grades	Undergraduate university students, Germany
Skilling, Stylianides	Cognitive engagement framework (Skilling & Stylianides, 2022) Self-determination theory (Ryan & Deci, 2020)	Engagement	Coding of responses based on items to video-vignettes	Teachers, UK

### Contributions anchored in control-value theory

Bieleke et al. (2022) collected data documenting the validity of the Achievement Emotions Questionnaire-Mathematics (AEQ-M), which measures enjoyment, pride, anger, anxiety, shame, hopelessness, and boredom in mathematics. The analysis supported the expected internal structure of the AEQ-M and its external relationships to learning outcomes. Putwain and Wood (2022) showed reciprocal negative relationships between control and anxiety. Although anxiety predicted lower value, value was unrelated to subsequent anxiety. Schubert et al. (2022) demonstrated that control and value appraisals were related to positive and negative emotions. Enjoyment and curiosity mediated the relationships between appraisals and students’ attention and motivation during the proof construction task.

### Contributions anchored in expectancy-value theory

Beswick et al. (2022) identified three motivational profiles in male students, namely, Positively Engaged, Disengaged, and Struggling Ambitious, which were differently predicted by mastery classroom goal structure, perceived peer valuing of mathematics, and teacher enthusiasm. Böswald and Schukajlow (2022) found specific differences between teachers’ ratings of their own and hypothetical students’ value and self-efficacy beliefs depending on the type of problem, as well as a positive correlation between teachers’ judgments of their own and their students’ value and self-efficacy. Gaspard et al. (2022) showed that effects of an intervention promoting utility value were larger when students perceived higher relevance support before and after the intervention. Jenifer et al. (2022) emphasized the role of anxiety by showing that highly math-anxious students reported allocating smaller proportions of their study time to problem-solving (ranked as the most effortful study strategy) compared with their less anxious peers.

### Contributions anchored in self-determination theory, interest theory, or well-being theory

Renninger et al. (2022) demonstrated that interest corresponds to and potentially scaffolds the comprehension of mathematical argumentation. Seah (2022) revealed that Chinese students valued perseverance for their mathematical well-being significantly more than other factors (e.g., mathematical engagement, relationships, or meaningfulness). Zhang et al. (2022) showed that motivation was positively related to cognitive engagement, and cognitive engagement was positively related to intrinsic motivation.

### Contributions anchored in multiple theories

Hettinger et al. (2022) showed that teacher self-efficacy for student engagement positively predicted student-perceived but not teacher-perceived emotional support, which in turn positively predicted students’ mathematics interest. With a longitudinal path analysis, Middleton et al. (2022) revealed that motivation predicted emotions in mathematics classes. Panero et al. (2022) analyzed the longitudinal development of math attitudes and demonstrated how different dimensions of these attitudes influence each other. Rach (2022) found that individual characteristics (e.g., interest) in university mathematics were strong predictors of task values. Task values fluctuated only slightly across different situations within students, and task values were positively related to effort, autonomy, and competence. The analysis of video vignettes by Skilling and Stylianides (2022) indicated that slightly more than half of the teachers favored a controlling style regarding students’ strategy use, whereas other teachers preferred a teaching style that promoted students’ autonomy in strategy use.

## Summary and future directions

Research on emotions and motivation has received growing attention in recent decades. In recent years, analyzing the development of relative numbers of papers in two mathematics journals from 2014 to 2021, we found a substantial increase in papers that included emotional and motivational variables. After a long period in which cognitive approaches were dominant, this development signals an *affective turn* in research on mathematics education. The increasing recognition of the importance of emotions and motivation is equivalent to similar developments in other disciplines, from the humanities to economics, psychology, and the neurosciences, during the past three decades. In recent years, additional drivers of this development may have had challenges (e.g., the COVID-19 pandemic or issues of social and racial diversity), which are more often addressed now in mathematics education research.

We hope that the trend toward acknowledging the relevance of emotions and motivation in mathematics education will continue in the future. The summary of concepts and theories of emotions and motivation and related intervention research that we provided in this overview may serve as a basis for future research. We view the following issues as critical for future research.

### Theory development

The conceptual overlap between constructs is an important characteristic of research on emotions and motivation (Di Martino & Zan, 2015; Hannula et al., 2019). It results from diversity in the theoretical approaches applied to investigate affective variables. It is crucial but challenging to overcome the resulting fragmentation that is typical in the field. We call for more theoretical and empirical work that addresses conceptual similarities and differences between constructs, such as self-concept and self-efficacy (Marsh et al., 2019), and contributes to their integration, with a specific focus on mathematics.

In our discussion of the overlap of constructs, we noted how researchers deal with sibling constructs, such as statistically controlling for a sibling in the analysis. As noted, such a covariate might pose a threat to validity because it can result in excluding relevant components of the target construct from measures of the construct. Careful consideration before controlling for variables, combining constructs rather than keeping them separate, and combining alternative analyses of findings with and without the exclusion of variables are possible strategies for avoiding such a fallacy.

For researchers working in the field of emotions and motivation, we recommend considering the following key characteristics of affective variables: *valence, temporal stability* (vs. variability), *situational specificity* (vs. generality), and *objects* of emotions and motivation. Valence and temporal stability (vs. variability) are well-known characteristics that are considered in many taxonomies in the field. The situational nature and objects of constructs have received increased attention recently. Addressing these characteristics might help clarify similarities and differences between constructs and contribute to structuring the field.

The structure of the objects of emotions and motivation and their relevance to explaining relationships with learning outcomes have not been sufficiently investigated and deserve more attention (with exceptions such as Goetz et al., 2006). Developing theoretical models of the hierarchical structure of the objects of emotions and motivation and of relationships between objects (e.g., topics, competencies, or strategies) may be important for advancing measurement and research in the field.

### Empirical paradigms: between-person and within-person research

To examine the causal mechanisms linking different emotional and motivational processes and their relationships with learning and achievement, it will be important to use within-person analytical paradigms. The field is currently dominated by studies inspecting between-person distributions of variables and the links between these distributions. This is true for both nonexperimental field studies and laboratory experiments. The former use the between-person covariation of variables to investigate their relationships, the latter between-subject experimental designs. A major problem with this approach is that between-person data are not suited for drawing conclusions about the within-person causal mechanisms that explain relationships between variables, except when specific conditions (e.g., ergodicity) that are rarely met (Murayama et al., 2017; Voelkle et al. 2014) hold. In field studies, the between-person and within-person covariation of variables can diverge widely (Hamaker et al., 2015; Orth et al., 2021). In between-subject experiments, differences between experimental conditions may mask differential effects for different groups of participants. As such, to elucidate the mechanisms that drive the effects of emotions and motivation on learning, future studies should focus on complementing between-person research with within-person study designs (e.g., for a within-person analysis of students’ emotions and learning outcomes, see Pekrun et al., 2023).

### Measures and dynamic processes

On a related note, we call for more research on differences between measures. To what extent do research findings depend on the type of assessment? For example, self-report items can refer to specific mathematical problems or to mathematics as a whole domain. The appropriate level of generality may depend on principles of construct symmetry and the outcomes to be predicted. Another question is, how can measures beyond self-reports contribute to the assessment of emotions and motivation? As the majority of theories emphasize the importance of individuals’ interpretation of external events for the generation of emotions and motivation, self-reports remain the primary method. However, alternative measures (e.g., analyses of facial expressions or parameters of physiological arousal) can contribute to a more comprehensive assessment of emotions and motivation during learning.

Challenges in the measurement of emotions and motivation are especially likely to arise in research with preschool and primary school children (Batchelor et al., 2019). However, research with young children is crucial for understanding the early development of emotions and motivation in mathematics, including the development of relations between emotions and motivation, on the one hand, and cognitive variables, on the other (Di Martino, 2018).

Dynamic processes of emotions and motivation and the interplay of situational factors and dispositional factors (traits) in shaping these processes have received growing attention recently and should be considered in future research. Experience-sampling methodology that allows researchers to collect data in multiple data collection waves during learning can contribute to this research.

### Considering diversity and inclusion

Testing and, if needed, revising theories from the perspective of marginalized student groups is another important critical point. Theories of emotions and motivation have primarily been developed and tested in samples from Western, educated, industrialized, rich, and developed (WEIRD) countries, whereas students from other sociocultural contexts have been underrepresented. Testing the validity of existing theories to explain emotions and motivation in students who are discriminated against because of their racial, cultural, or social belonging is an open avenue for future research (for an example, see the study with Chinese students by Zhang et al., 2022).

### Synthesis of empirical findings

With a few recent exceptions (e.g., Barroso et al., 2021), we see a lack of contributions that have offered a synthesis of empirical findings in the domain of mathematics. In the past, syntheses of empirical findings have often been undertaken without differentiating between domains (e.g., Tze et al., 2016; Wigfield et al., 2021). We need syntheses of findings for research on emotions and motivation in mathematics education specifically. Meta-analyses and systematic reviews are essential for progress in the field given the increasing numbers of studies on affective variables.

### Intervention research and changing educational practices

Finally, our review of intervention studies identified promising instructional approaches that can promote beneficial emotions or motivation. However, the findings are partly inconsistent, thus indicating a strong need for replication studies and for identifying boundary conditions for positive effects of teaching approaches. Prior research has revealed that individual differences, such as students’ gender, prior knowledge, or initial levels of affect (Kosovich et al., 2019; Schukajlow et al., 2019), as well as contextual factors, such as social norms, learning content, duration of the intervention, or the quality of engagement during the intervention (Higgins et al., 2019; Liebendörfer & Schukajlow, 2020; Rosenzweig et al., 2022) might influence the effectiveness of the teaching approaches. There is a need for research to systematically investigate the roles of possible moderators and related methods to personalize interventions. Furthermore, there is a need for research that can transcend the intervention approach and explore how affectively sound changes in educational practices can be implemented in the mathematics classroom and in educational institutions more generally on a large scale. For example, researchers should investigate how instructional settings and practices can support students in regulating their emotions and motivation in ways that promote their development.
